# Pre-Clinical Studies of Inactivated Polyvalent HFRS Vaccine

**DOI:** 10.3389/fcimb.2020.545372

**Published:** 2020-11-02

**Authors:** Tamara K. Dzagurova, Alexandra A. Siniugina, Aidar A. Ishmukhametov, Maria S. Egorova, Svetlana S. Kurashova, Maria V. Balovneva, Andrey A. Deviatkin, Petr E. Tkachenko, Oksana A. Leonovich, Evgeny A. Tkachenko

**Affiliations:** ^1^ Chumakov Federal Scientific Center for Research and Development of Immune-and-Biological Products Russian Academy of Science, Moscow, Russia; ^2^ Institute for Translational Medicine and Biotechnology, Sechenov First Moscow State Medical University, Moscow, Russia; ^3^ Institute of Molecular Medicine, Sechenov First Moscow State Medical University, Moscow, Russia; ^4^ Department of Internal Medicine Propaedeutics, Sechenov First Moscow State Medical University, Moscow, Russia

**Keywords:** hemorrhagic fever with renal syndrome, vaccine, immune response, Puumala, Hantaan, Dobrava-Belgrad ortohantaviruses

## Abstract

Hemorrhagic fever with renal syndrome (HFRS) is the most common natural focal disease in the Russian Federation with about 6–12 thousand cases annually. 97.7% of all HFRS cases in Russia are caused by the Puumala virus, 1.5%—by the Hantaan, Amur, Seoul viruses, and about 0.8% by the Kurkino and Sochi viruses. There are no licensed vaccines for the prevention of HFRS in the European Region; there are no specific therapeutic to treat orthohantavirus infections. Here we report the results of candidate polyvalent HFRS vaccine preclinical studies. The vaccine was produced on the basis of three viruses: Puumala, strain PUU-TKD/VERO, Hantaan, strain HTN-P88/VERO, and Sochi, strain DOB-SOCHI/VERO. These viruses were inactivated with β-propiolacton, purified by gel filtration and aluminum hydroxide adsorbed. 18–20 g female BALB/c mice were immunized intramuscularly 2 or 3 times with a 2-week intervals and blood was taken 2 weeks after immunization. FRNT_50_ performed for virus specific antibodies determination. ELISA kits (Bender MedSystems, Cusabio) were used for detection of cytokines IL-1β, IL-12, INF-ɣ. Neutralizing antibodies geometric mean titers to the Puumala, Hantaan, and Sochi viruses were: 9.22 ± 0.31, 9.17 ± 0.26, 8.96 ± 0.34 log_2_/ml. Up to 1/32 vaccine dilution neutralizing antibodies were identified in 10/10 immunized mice with titers ≥ 3,32 log_2_/ml. IL-12 and INF-ɣ increased after immunization in average 5.5 and 2.8 times respectively, that reflects the Th1 type immunity stimulation. IL-1β slightly increased, that may suggest vaccine low reactogenicity. According to our preclinical investigations, the candidate polyvalent HFRS vaccine elicits balanced immune response to the Puumala, Hantaan and Sochi viruses.

## Introduction

Hemorrhagic fever with renal syndrome (HFRS) is a non-transmissible viral zoonosis widespread in Eurasia. In Russia HFRS is one of the most important natural focal human diseases (Tkachenko et al., 2019). The causative agents of HFRS are the *orthohantaviruses Puumala*, *Seoul, Hantaan* (Hantaan, Amur, Suchong viruses), *Dobrava-Belgrade* (Dobrava, Sochi, Kurkino, and Saaremaa viruses)^1^. In Russia, HFRS is caused by Puumala (PUUV), Hantaan (HTNV), Seoul, Kurkino, and Sochi (SOCHI) viruses (Tkachenko et al., 2019). HFRS cases are registered in 68 out of 85 regions of the Russian Federation and annually comprise 6,000–11,000 patients^2^. According to Federal Service for Surveillance on Consumer Rights Protection and Human Wellbeing Statistical materials 2000–2018^2^ for the period from 2000 to 2018 137,430 HFRS cases were registered, including 3300 children under the age of 14 years. In 570 patients with HFRS, the disease was fatal. More than 98% of HFRS cases were detected in the European part, the vast majority of which (more than 98%) caused by the Puumala virus (Tkachenko et al., 2019). In the late 1990s, in the forest-steppe zone of the central regions of Russia, the Kurkino virus foci (the genetic variant of the Dobrava-Belgrade orthohantavirus) were established (Tkachenko et al., 2005a). Along with annual sporadic cases, three HFRS-Kurkino outbreaks with about 1,200 cases in total were recorded (Klempa et al., 2008). In the early 2000s, in the Krasnodar Territory, a new genotype of the *Dobrava-Belgrade orthohantavirus*—Sochi virus (SOCHIV) has been identified. Sporadic incidence of HFRS-Sochi characterized by severe clinical course and mortality up to 14% (Tkachenko et al., 2005b; Kruger et al., 2015). In the Asian part, mainly in the Far East, about 90–120 HFRS cases (about 1.6 per 100,000) caused by the Hantaan, Amur, Seoul viruses are registered annually. Epidemiological analysis showed that 97.7% of all cases in Russia are etiologically caused by the Puumala virus, about 0.8% by the Kurkino and Sochi viruses, 1.5% by the Hantaan, Amur, and Seoul viruses (Tkachenko et al., 2019). Anti-epidemic measures in HFRS are mainly aimed to limit the contacts of people with rodents. Despite ongoing preventive measures, the incidence of HFRS in active foci does not tend to decrease (Tkachenko et al., 2016). Vaccination appears to be the most effective way to control this infection. Preventive vaccination has been successfully used in China and Korea for over 20 years (Sohn et al., 2001; Wang et al., 2012), however, none of these vaccines can be used in the European regions, since all of them are based on the Hantaan and Seoul viruses and do not have a protective effect against the Puumala virus, the main causative agent of HFRS in Russia. Various experimental vaccines against PUUV have been developed, including inactivated virus vaccines (Lee et al., 1999), virus-like particles (Ulrich et al., 1999), and subunit vaccines. Most of the subunit vaccines have involved the N protein produced in *Escherichia coli*, yeasts (Dargeviciute et al., 2002; de Carvalho Nicacio et al., 2002), mammalian cells (Kallio-Kokko et al., 2001), and chimeric hepatitis B virus core particles (Ulrich et al., 1998). These attempts to produce vaccines against PUUV were unsuccessful.

Difficulties with the cell culture derived vaccine against the PUUV were unsolved for a long time, mainly due to the difficulties in obtaining a high yield of this virus in cell cultures.

Certification of vaccine strains (PUU-TKD/VERO, DOB-EAT/VERO) in accordance with international requirements was successfully carried out follow up adaptation of the Puumala and Kurkino virus strains to Vero cells. Optimization of virus concentration, purification and inactivation methods, as well as specific control methods development have allowed to create a technology of generating a bivalent vaccine against HFRS based on the Puumala and Kurkino viruses in 2010 (Barkhaleva et al., 2011; Tkachenko et al., 2015). Subsequently a polyvalent formalin inactivated HFRS vaccine based on the PUUV, Hantaan (HTNV), and Sochi (SOCHIV) viruses has been created according to this technology (Sinyugina et al., 2019). The purpose of this study is to present the results of β-propiolacton inactivated polyvalent HFRS vaccine immunogenicity preclinical studies.

## Materials and Methods

**Polyvalent vaccine against HFRS** (hereinafter referred to as the vaccine) was produced on the basis of Puumala (strain PUU-TKD/VERO), Hantaan (strain HTN-P88/VERO), and Sochi (strain DOB-SOCHI/VERO) viruses propagated in Vero cells. Virus stocks were inactivated with β-propiolacton in a final dilution of 1:6000 (0,17 μl/ml). Completeness of virus inactivation was assessed in three consecutive passages of the vaccine semi product through the Vero cell culture followed by virus indication by means of IFA and FFU. Virus was concentrated by centrifugation in the tangential flow after clarification filtration, followed by purification on the Sepharose 6FF and Capto Сore 700 sorbents by means AKTA purifier chromatograph and C series columns (GE Healthcare). VAC dose was calculated for each virus according to the number of RNA copies/ml.

GenBank accession numbers of vaccine strains sequences are:

HTN-P88/VERO BankIt2108429: S-MH251328, M-MH251329, L-MH251330;

PUU-TKD/VERO BankIt2108429: S-MH251331; M - MH251332; L- MH251333;

DOB-SOCHI/VERO BankIt2108429: S-MH251334; M- MH251335; L- MH251336.

One ml of vaccine contains RNA copies: PUU-TKD/VERO – 2.8 ± 0.3 × 10^5^, HTN-P88/VERO – 3.6 ± 0.4 × 10^4^ and DOB-Sochi/VERO—1.8 ± 0,5 × 10^4^. The total protein content 52 µg/ml (by Lowry’s method).

Additional substances: solvent—phosphate buffer*, human albumin—1 mg, aluminum hydroxide (1.0 mg); cellular DNA < 10 ng/ml (by RT-PCR).

*buffer salts: sodium chloride (Eur.Ph.)—4 mg/ml; potassium chloride (Eur.Ph.)—0.1 mg/ml, sodium phosphate (Eur.Ph.)—0.71 mg/ml; potassium phosphate (Eur.Ph.)—0.12 mg/ml.

### Bioethics and Biosafety

Animal studies were carried out in accordance with the ethical principles established by the European Convention for the Protection of Vertebrate Animals used for experimental and other scientific purposes approved in Strasbourg on 03/18/1986 and confirmed in Strasbourg on 06/15/2006. The Protocol of animal studies was approved by the Ethics Committee of the Chumakov Federal Scientific Center for Research and Development of Immune-and-Biological Products of Russian Academy of Sciences. Biohazard experiments, including live viruses and cells, were conducted in a laboratory under Biosafety Level 3 (BSL-3).

### Immunization

Pathogen-free female BALB/c mice weighing 18–20 g at the beginning of the study were purchased from the Andreevka branch of the Federal State Institution of Science “Scientific Center for Biomedical Technologies of the Federal Medical and Biological Agency” of Russia. Mice were kept in accordance with the laboratory animal control guidelines and were housed in the same conditions of maintenance and feeding in the facility of the Chumakov Federal Scientific Center for Research and Development of Immune-and- Biological Products of Russian Academy of Sciences. Animals meeting the criteria for inclusion in the experiment were divided into groups of 10 mice. To exclude the influence of the researcher preferences on the experimental groups formation, the animals were seated by random selection. The experimental mice were injected intramuscularly with 0.5 dose of the vaccine (0.5 ml) undiluted and in dilutions of ^1^/_2_, ^1^/_8_ and ^1^/_32_ two or three times at two-week intervals. Mice of the control group (C) were injected with the same volume of 0.85% sodium chloride solution with the corresponding additives contained in the vaccine. Injection of 0.5 ml fluid was accompanied by a slight swelling of the muscle, which disappeared within 15–20 min. Blood was taken from eyes of animals before and 2 weeks after second or third immunizations. Serum samples were stored at 6 ± 2°C or for not more than a week. A portion of serum aliquots was stored at -80°C for not more than a month, and then tested for neutralizing antibodies and cytokines for 24 h.

### Cell Culture

Vero Е6 (ATCC^®^ No. CRL-1586) cell culture used for virus titer and neutralizing antibodies determination.

### Viruses

PUUV, strain K-27/Ufa-85 (Dzagurova et al., 2008), HTNV, strain 76/118 (Lee et al., 1978), SOCHIV, strain Ap1595/Sochi-01 (Tkachenko et al., 2005b).Viruses were stored at -70^о^C as aliquots of infected Vero E6 cell culture supernate.

Immune blood sera. Convalescent blood sera of HFRS-PUU, HFRS-HTN, HFRS-Sochi patients. Experimental rabbit blood sera to strains K-27/Ufa-85, 76/118, Ap1595/Sochi-01.

### Virus Titration

The viral stocks, prepared from the infected Vero E6 cells supernats, were titrated in 24-well plates by the focus forming units (FFU) assay us described earlier (Dzagurova et al., 2008). Briefly, the monolayer of Vero-E6 cells grown in the 24-well plate were incubated with 10-fold dilutions of virus samples for 1 h. Hereinafter, Eagle’s MEM was used as a diluent. Then the inoculum was removed and Eagle’s medium, containing 10% fetal bovine serum and 0.4% or 0.6% methylcellulose was added. After 6–7 days of incubation at a 37 ± 1^о^C FFU were visualized by immunofocus technique. Methylcellulose medium was removed from the wells and the cell monolayer was washed with 0.9% NaCl solution. 96° ethanol cooled to 4 ± 2°С was added for 15 min at 18–25°С in to the plate wells. After removing the fixative, the wells were washed with PBS and 200 μl of specific anti-hantavirus antibodies were added. For this purpose, the convalescent blood sera of HFRS-PUU, HFRS-HTN, HFRS-Sochi patients with IFA titers 1:64000 in dilution 1:200 were used. After 1 h incubation at 37 ± 1°C, the wells were washed 3 times for 5 min with 0.9% NaCl solution and 200 μl of protein A peroxidase conjugate was added to each well. After 1 h of exposure at 37 ± 1°C, the wells were washed and the DAB/NiCl_2_ indicator was added. 15–20 min later the wells were washed with running water, dried, and the spots (FFU) were counted visually. The virus titer expressed in lg FFU/ml.

### Focus-Reduction Neutralization Test_50_

FRNT_50_ performed as previously described (Dzagurova et al., 2008). Briefly, serum samples were diluted serially in 2-fold steps beginning with 1:10 dilution, mixed with an equal volume of the corresponding virus containing 50–100 focus-forming units and incubated for 1 h at 37°C or overnight at 4 ± 2°C for neutralization of virus. In control wells test dose of the virus was incubated with Eagle’s MEM alone or non-immunized mice sera. Then virus-serum mixture was inoculated onto the confluent Vero E6 cells. After 6–7 days of incubation FFU were detected as described above. Neutralizing antibody titer was expressed as the reciprocal of highest serum dilution that resulting in greater than 50% reduction in the number of FFU relative to the average number of FFU in control wells. Each blood serum sample was three times tested in FRNT_50_. NAb titer in the control mice did not exceed 2.32 log_2_ (the limit of clipping). The criterion of sufficient immunogenicity was considered neutralizing antibody titer ≥ 4.32 log_2._

### Cytokines Quantification

The cytokines estimation in mouse blood was carried out two weeks after immunization. Cytokines: IL-1β, IL-12, IFN-ɣ were determined by means of ELISA kits (Bender MedSystems, Cusabio) in accordance with the manufacturer’s protocol. The assay employs the quantitative sandwich enzyme immunoassay technique. Antibody specific for **IL** (a certain kind) has been pre-coated onto a microplate. Standards and samples are pipetted into the wells and **IL** present is bound by the immobilized antibody. After removing any unbound substances, a biotin-conjugated antibody specific for **IL** is added to the wells. After washing, avidin conjugated Horseradish Peroxidase is added to the wells. Following a wash to remove any unbound avidin-enzyme reagent, a substrate solution is added to the wells and color develops in proportion to the amount of **IL** bound in the initial step. The color development is stopped and the intensity of color is measured. The results are presented in pg/ml using the Curve Expert program. Detection range—15.625–1000 pg/ml.

### Statistics

Neutralizing antibodies detection results (samples from individual mouse obtained in three independent experiments) were analyzed in GraphPad Prism version 8.3.1 (La Jolla, CA, United States). All experiments were performed independently at least three times. The statistical significance of the differences was determined using the one-way ANOVA with the test of Tukey’s and Dunnett’s multiple comparisons: ns = not significant, * p <0.05, ** p <0.01, *** p <0.005, **** p <0.0001. The results were presented as the average geometric values of the NAb in binary logarithms. Cytokines were quantified using CurveExpert software.

## Results

The vaccine immunogenicity was monitored by the induction of neutralizing antibodies, as well as by certain cytokines in response to BALB/c mice immunization.

For neutralizing antibodies detection PUUV, HTNV, and SOCHIV test-strains were used in FRNT_50_ experiments.

The type-specificity of the vaccine and test-strains of the PUUV, HTNV, and SOCHIV assessed by the cross-neutralization (**Table 1**). In accordance with these data, the vaccine and test-strains of the PUUV, HTNV, and SOCHIV do not differ from each other more than two times in immune sera antibodies titer, confirming their serological identity.

**Table 1:** Type specificity of the vaccine and test strains PUUV, HTNV, and SOCHIV.

Virus	Strain	FRNT_50_					
		PUU-314*	PUU-R4^1)^	HTN-3316*	HTN-R1^3)^	Sochi-1334*	Sochi-R6^2)^
PUUV	PUU-TKD/Vero	32768	640	<20	<20	<20	<20
	К-27/Ufa-85	16384	640	<20	<20	<20	<20
HTNV	HTN-Р88/Vero	<20	<20	8192	320	40	<20
	76/118	<20	<20	8192	320	20	<20
SOCHIV	DOB-Sochi/Vero	<20	<20	0	<20	8192	320
	Ар1595/Sochi-01	<20	<20	0	<20	8192	640

*Convalescent blood sera HFRS-PUU, HFRS-HTN, HFRS-Sochi; experimental rabbit blood sera to strains: 1) K-27/Ufa-85; 2) 76/118; and 3) Ap1595/Sochi-01.

**Figure 1:**
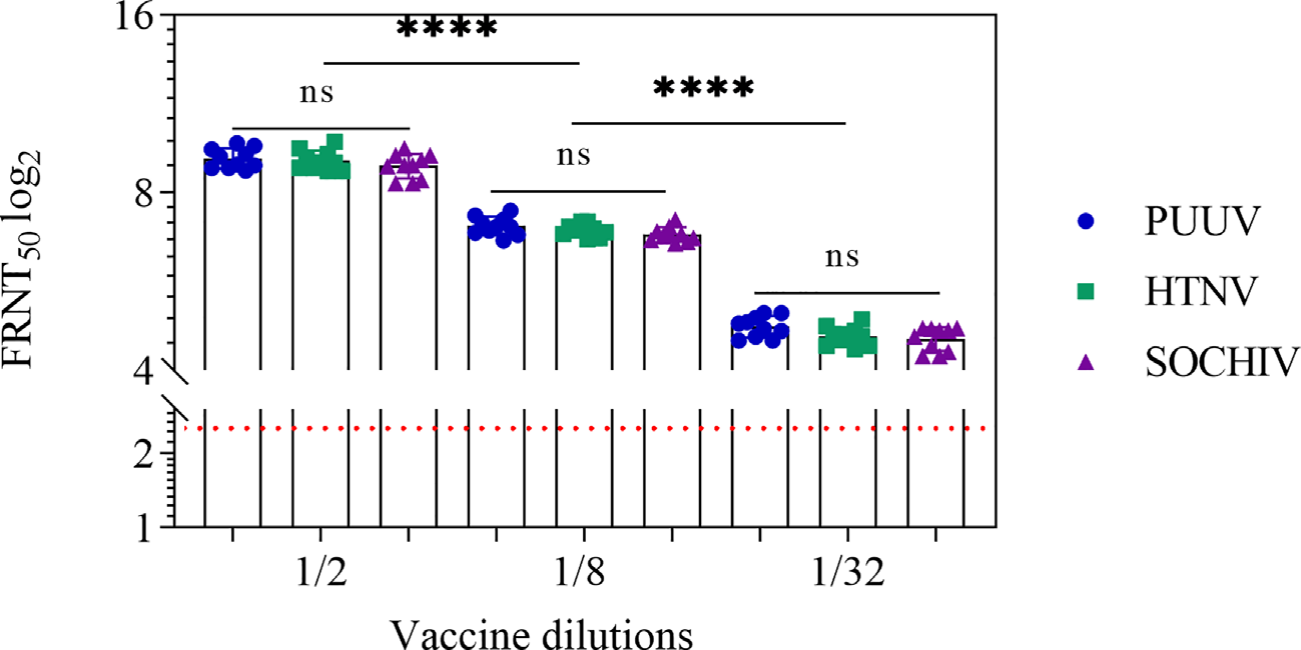
Neutralizing antibodies in the BALB/c mouse sera 2 weeks after second immunization. The sera collected from the mice immunized with vaccine in dilutions. Neutralizing antibody titers for individual mice were shown. The FRNT_50_ limit of NAb detection is a titer of 2.32 log_2_ (dashed line). Statistic analysis was performed using a one-sided ANOVA with Tukey’s multiple comparisons test: ns, not significant, ****p < 0.0001.

The immune response after BALB/c mice immunization was monitored 2 weeks after 2^nd^ and 3^rd^ immunizations. After two immunizations, NAb were detected in the sera of 10 out of 10 mice against each of three viruses up to 1/32 vaccine dilution. In the ½ vaccine dilution the average titer of PUUV NAb was 9.2 ± 0.4 log_2_, HTNV—9.1 ± 0.4 log_2_, SOCHIV—8.9 ± 0.6 log_2_. The NAb titers decreased in proportion to the vaccine dilution (**Figure 1**).

**Figure 2:**
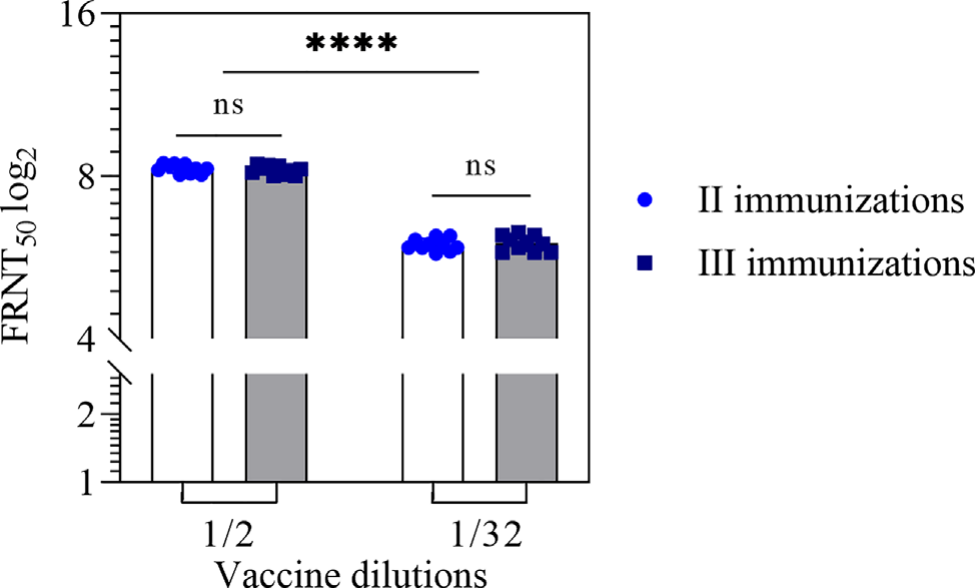
Neutralizing antibodies titer in the mouse sera after two and three immunizations with the vaccine in 1/2 and 1/32 dilutions. NAb titer for individual mice were shown. Statistical analysis was performed using a one-sided ANOVA with Tukey’s multiple comparisons test. ns, not significant; ****p < 0.0001.

An increase in the number of immunizations up to 3 times at 2-week intervals did not lead to an increase in the neutralizing antibodies titer (**Figure 2**).

**Figure 3:**
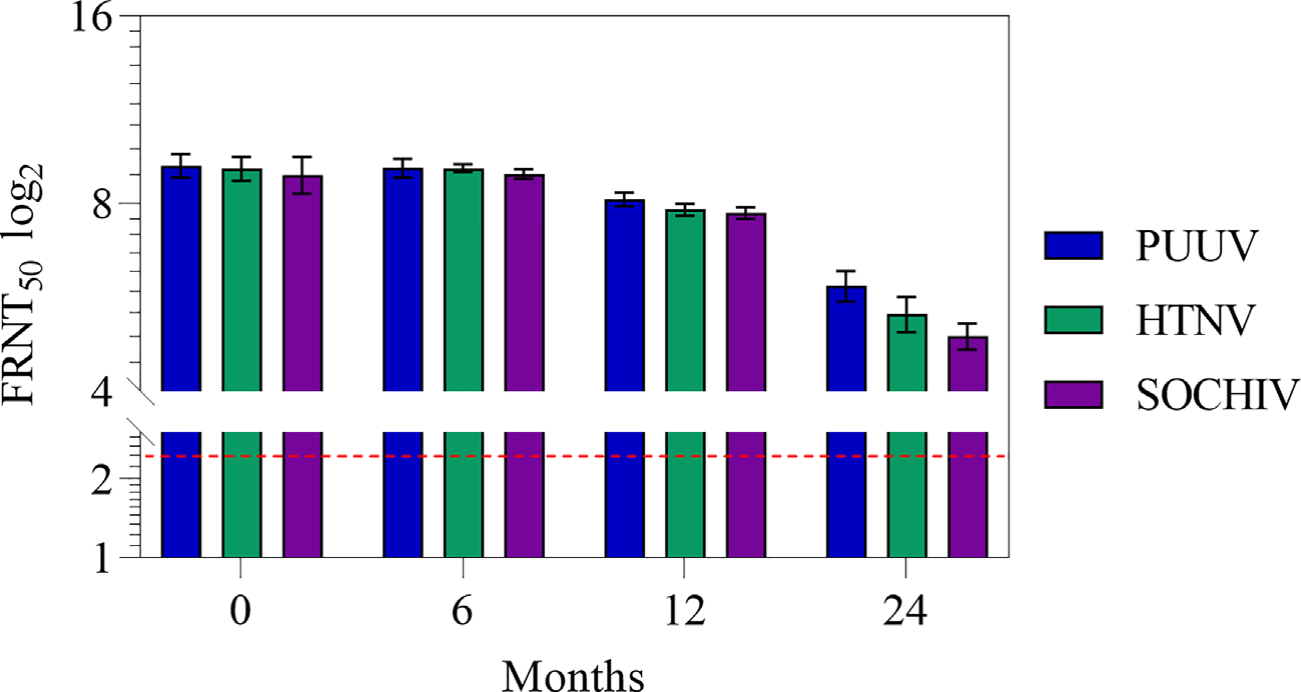
Antibodies to the PUUV, HTNV and SOCHIV after two immunizations of mice with the vaccine during storage in dilutions: А- 1/2. The FRNT_50_ limit of detection was a NAb titer of control group 2.32 log2 (dashed line). Sera were tested in FRNT_50_. The mean values and standard deviations for each group of 10 mice were given in the graph. Statistic analysis was performed using a one-sided ANOVA with Tukey’s multiple comparisons test.

Stability of the vaccine was determined after 6, 12, 24 months of storage by testing immunogenicity (**Table 2**, **Figure 3**). After 6 months storage, NAb titers to the PUUV, HTNV, and SOCHIV remained the same as 2 weeks after immunization. After 12 months storage of the vaccine NAb decreased 2 times to the PUUV, 2.5 times to the HTNV, and 3 times to SOCHIV in average. Twenty-four months later, the antibody titers decreased approximately 9, 13, and 16 times to PUUV, HTNV, and SOCHIV accordingly. Nevertheless, the vaccine in dilution 1/32 induced the NAb production in 10/10 mice achieving a titer of at least 1/20.

**Table 2:** Immunogenicity of the vaccine stored under controlled conditions (6 ± 2°C).

Storage time	Vaccine dilution	Antibody positive/total mice			FRNT_50_ antibody titer, log2		
		PUUV	HTNV	SOCHIV	PUUV	HTNV	SOCHIV
2 weeks	1/2	10/10	10/10	10/10	9.22 ± 0.31	9.17 ± 0.26	8.96 ± 0.34
	1/8	10/10	10/10	10/10	7.1 ± 0.17	7.5 ± 0.21	6.85 ± 0.3
	1/32	10/10	10/10	10/10	4.73 ± 0.2	4.5 ± 0.16	4.75 ± 0.1
6 months	1/2	10/10	10/10	10/10	9.15 ± 0.3	9.12 ± 0.13	8.98 ± 0.2
	1/8	10/10	10/10	10/10	7.3 ± 0,13	6.8 ± 0.19	6.97 ± 0.2
	1/32	10/10	10/10	10/10	4.57 ± 0.19	4.6 ± 0.2	4.76 ± 0.2
1 year	undiluted	10/10	10/10	10/10	9.05 ± 0.2	8.95 ± 0.19	8.87 ± 0.3
	1/2	10/10	10/10	10/10	8.17 ± 0.18	7.87 ± 0.2	7.8 ± 0.29
	1/8	10/10	10/10	10/10	6.9 ± 0.23	6.8 ± 0.19	6.56 ± 0.3
	1/32	10/10	10/10	10/10	4.7 ± 0.25	4.5 ± 0.2	4.48 ± 0.17
2 years	undiluted	10/10	10/10	10/10	7.2 ± 0.19	6.3 ± 0.22	6.09 ± 0.24
	1/2	10/10	10/10	10/10	5.94 ± 0.3	5.3 ± 0.29	4.9 ± 0.24
	1/8	10/10	5/10	5/10	5.5 ± 0.2	4.91 ± 0.3	4.8 ± 0.26
	1/32	10/10	10/10	10/10	4.1 ± 0.18	3.79 ± 0.2	3.7 ± 0.26
Control mice							
2 weeks	undiluted	0/10	0/10	0/10	<2.32*	<2.32	<2.32
6 months	undiluted	0/10	0/10	0/10	<2.32	<2.32	<2.32
1 year	undiluted	0/10	0/10	0/10	<2.32	< 2.32	<2.32
2 years	undiluted	0/10	0/10	0/10	<2.32	<2.32	<2.32

*The cut-off limit.

**Figure 4:**
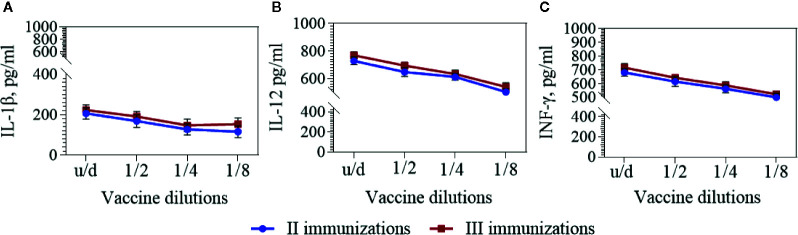
IL-1β **(A)**, IL-12 **(B),** and IFN-γ **(C)** in the BALB/c mouse sera. Cytokines were detected by means commercially available ELISA kits 2 weeks after second and third immunizations with the vaccine in dilutions. The mean values and standard deviations for each group of 10 mice were given in the graph. Statistic analysis was performed using a one-sided ANOVA with Kolmogorov-Smirnov test.

To assess the cytokine immune response, interleukins IL-1β, IL-12, and INF-ɣ levels in the BALB/c mouse sera was measured before and 2 weeks after the second and third immunizations. The cytokine levels in intact mice prior to immunization were taken as the background level. The quantitative content of these cytokines after two and three immunizations with a vaccine in dilutions had no statistically significant differences (**Figure 4** and **Table 3**).

**Table 3:** IL-1β, IL-12, and IFN-γ in the BALB/c mouse sera 2 weeks after second and third immunizations with the undiluted vaccine.

Cytokines	0*	II immunizations	III immunizations
IL-1β	150.5 ± 18**	206.4 ± 28	223.6 ± 27
IL-12	133 ± 15	729.4 ± 28	769.6 ± 25
IFN-γ	248 ± 23	681.6 ± 28	717.4 ± 28

*Cytokines level before immunization; **pg/ml.

In response to vaccine immunization, a slight (1.4–1.5 fold) increase in the number of IL-1β cytokines circulating in the blood of BALB/c mice was observed two weeks after the second and third immunizations. At the same time, 2 weeks after the second and third immunizations the number of IL-12 cytokines increased 5.5–5.8 times and INF-ɣ—2.7–2.9 times correspondingly.

## Discussion

Among the pathogenic hantaviruses, only for the Andes virus there is known a laboratory animal - golden hamsters, which completely reproduces the infection that occurs in humans (Safronetz et al., 2012). For the viruses Puumala, Hantaan and Sochi there is no such a model yet. These viruses multiply asymptomatically both in their main hosts (bank voles, field mice and Caucasian forest mice, respectively) and in a laboratory animals (mice, rats, hamsters) (Bernshtain et al., 2010). Pathogenic virus is excreted with saliva, urine and for a long time remains viable in the environment which correspondingly requires compliance with Biosafety Level 3 conditions in the vivarium. These circumstances greatly complicate the testing of HFRS vaccines by means challenge. In this regard to simplify the HFRS vaccines immunogenicity control, we used quantitation of the neutralizing antibodies, which are known as the determinants of immune protection along with T-cell immunity.

BALB/c mice immunization showed that the polyvalent vaccine induced neutralizing antibodies equally to PUUV, HTNV, and SOCHIV. The antibody titers in the mouse sera were high enough for all the targeted viruses. The content of each immunogen (inactivated whole virus) in the vaccine did not differ significantly, which can be judged by the amount of viral RNA/dose. This composition of immunogens in vaccine provided a balanced immune response in BALB/c mice.

Vaccine stability investigations showed that neutralizing antibody titers induced to the PUUV, HTNV, and SOCHIV do not change until 6 months of storage and slightly decreases after 12 months of storage. After 24 months storage, neutralizing antibody titer to these viruses decreased approximately 9, 13, and 16 times respectively. Nevertheless, NAbs were detected even in 1/32 vaccine dilution in 10/10 mice with titers of at least 1/20. The decrease in vaccine immunogenicity during storage is inherent in all inactivated vaccines and motivate the use of various stabilizers and adjuvants. We have obtained encouraging results of enhancing the immunogenicity of the vaccine after long-term storage when using low-toxic lipopolysaccharide as an adjuvant (unpublished data).

It is known that T-cell mediated immunity has an important role in antiviral protection. To assess the stimulation of T-cell immunity, we studied the cytokines IL-12 and IFN-γ in the serum of immunized mice 2 weeks after the second and third immunizations. IL-12 is secreted by monocytes/macrophages, dendritic cells, B-cells (Ketlinsky and Simbirtsev, 2008) directs the specific immunity development according to the corresponding Th1 phenotype, and also stimulates the proliferation of natural killers (Trinchieri, 1995; Zhu et al., 2009). Induction of the IFN-γ synthesis by leukocytes, monocytes, fibroblasts and other cells inhibits virus replication, increases the expression of MHCs I and II and activates macrophages. Results of the IL-12 and IFN-γ quantifications in the mouse sera indicate a pronounced activation of these immune system effectors due to the vaccination.

As for IL-1β cytokine, which plays an important role in early innate immune responses, dysregulation of its activity leads to auto-inflammatory manifestations (Veerdonk and Netea, 2013; Palomo et al., 2015) and therefore excessive induction of IL-1β during vaccination is undesirable. In our experiments, there was a slight increase in the level of this cytokine 2 weeks after immunization, which may suggest of low grade reactogenicity of the vaccine candidate.

Since we did not study the dynamics of cytokines from the 1^st^ days post immunization, the data presented demonstrate the cytokine status only 2 weeks after immunization. It is possible that in the first days after vaccination, their number could be higher (or lower)?. For example, Mingala et al. (2009) showed that IFN*γ*, IL-10, and TNFa had peaked on week 3 in response to inactivated foot-and-mouth disease (FMD) vaccination while the remaining cytokines (IL-2, IL-4, IL-12p40) peaked on the 2nd week and decreased by the 3^rd^ week. The study of Sharma et al. (2018) presented results of immune response to FMD virus in clinically and subclinically infected adults and calves. IFN-γ and IL-21 expression was assayed on 0, 7, 14, 28, 60, 90, and 120 days post outbreak (DPO) by ELISA. The peak of IFN-γ expression was observed on 14 DPO across all categories of animals. The IL-21 level increased significantly during 14–28 DPO and highest expression was noticed on 28 DPO. In our study, we can compare cytokines IL-1β, IL-12, and IFN-γ activation degree 2 weeks after immunizations. The smallest increase was found for IL-1β (1.4–1.5 times), followed by IFN-γ (2.7–2.9 times) and IL-12 (5.5–5.8 times). It should be noted that these indicators of cytokines, most likely, are not indicators of their maximum increase after immunization.

It has been shown that the cytokines levels in the mice blood sera did not increased statistically significant after three immunizations compared with two immunizations, as it was in the case of neutralizing antibodies detection. These results make it possible to recommend two immunizations both for monitoring vaccine stability and for conducting future clinical trials. A third immunization may be needed as a booster and the time set could be revealed according to the NAbs persistence.

Thus, we can conclude that the polyvalent vaccine against HFRS, according to our study is able to induce a balanced immune response to the PUUV, HTNV, and SOCHIV. To overcome vaccine instability during storage (under 6 ± 2°C), it is planned to replace the aluminum hydroxide with apyrogenic low-endotoxic lipopolysaccharide (LPS) from *Shigella sonnei*. As it has been shown for the LPS-enhanced PUUV vaccine, immune response in BALB/c mice remained stable after 1 year of storage, and even with a vaccine dose reduction of 8 times, NAb titers did not decrease (unpublished data).The structural analog of LPS from *Shigella flexneri* 2a successfully passed clinical trials as candidate vaccine against *S. flexneri* 2a infection (Ledov et al., 2019). For polyvalent HFRS vaccine enhanced with LPS additional preclinical trials will be required.

## Data Availability Statement

The datasets presented in this study can be found in online repositories. The names of the repository/repositories and accession number(s) can be found below: https://www.ncbi.nlm.nih.gov/genbank/, BankIt2108429: S-MH251328, M-MH251329, L-MH251330.

## Ethics Statement

The animal study was reviewed and approved by The Protocol of animal studies was approved by the Ethics Committee of the Chumakov Federal Scientific Center for Research and Development of Immune-and- Biological Products of Russian Academy of Sciences.

## Author Contributions

ET and TD developed the original idea. ET, TD, and SK designed the whole study. AI and AS solved organizational issues. TD, SK, ME, MB, OL, and AD were involved in candidate vaccine preparation. SK, ME, MB, and OL performed the experiments. KS performed a statistical analysis. TD, ET, and PT drafted the manuscript. All authors contributed to the article and approved the submitted version.

## Conflict of Interest

The authors declare that the research was conducted in the absence of any commercial or financial relationships that could be construed as a potential conflict of interest.

## Footnotes

1
http://ictvonline.org/virusTaxonomy.asp

2
https://www.rospotrebnadzor.ru/activities/statistical-materials
